# Supplementary material for: Crystal Structure of the Epo1-Bem3 Complex for Bud Growth
Source: Int J Mol Sci. 2021 Apr 7;22(8):3812. doi: 10.3390/ijms22083812 (PMC8067709; doi:10.3390/ijms22083812)
Supplement: Supplementary file 1 [file ijms-22-03812-s001.pdf]

Supplementary Figures S1–S2

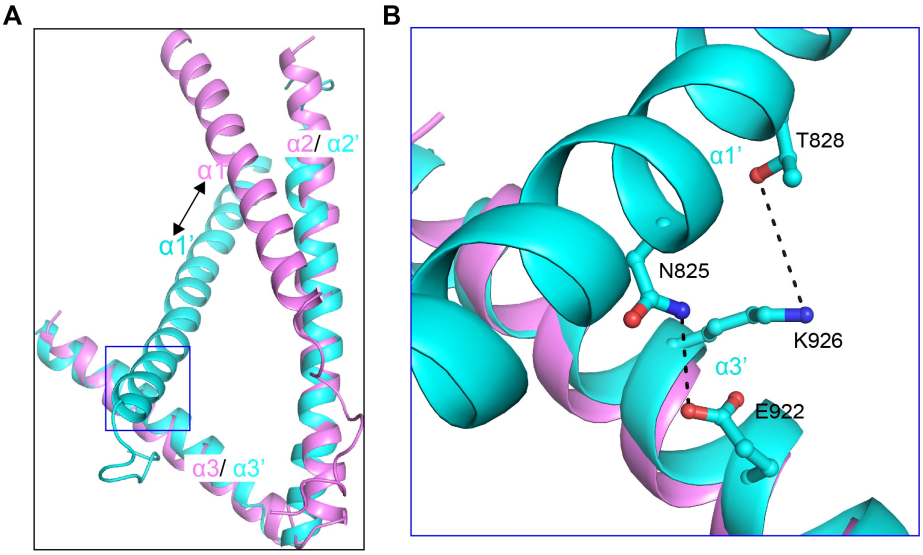

Figure. S1 Comparison two promoter of Epo1$^{CC2-CC4}$.

A, Superposition of Epo1$^{CC2-CC4}$ with non-crystallographic Epo1$^{CC2-CC4}$, show α1 and α1$^{'}$ have conformation changes. **B**, this change were maintains by two hydrophilic bonds, from N825 and E922, T828 and K926.

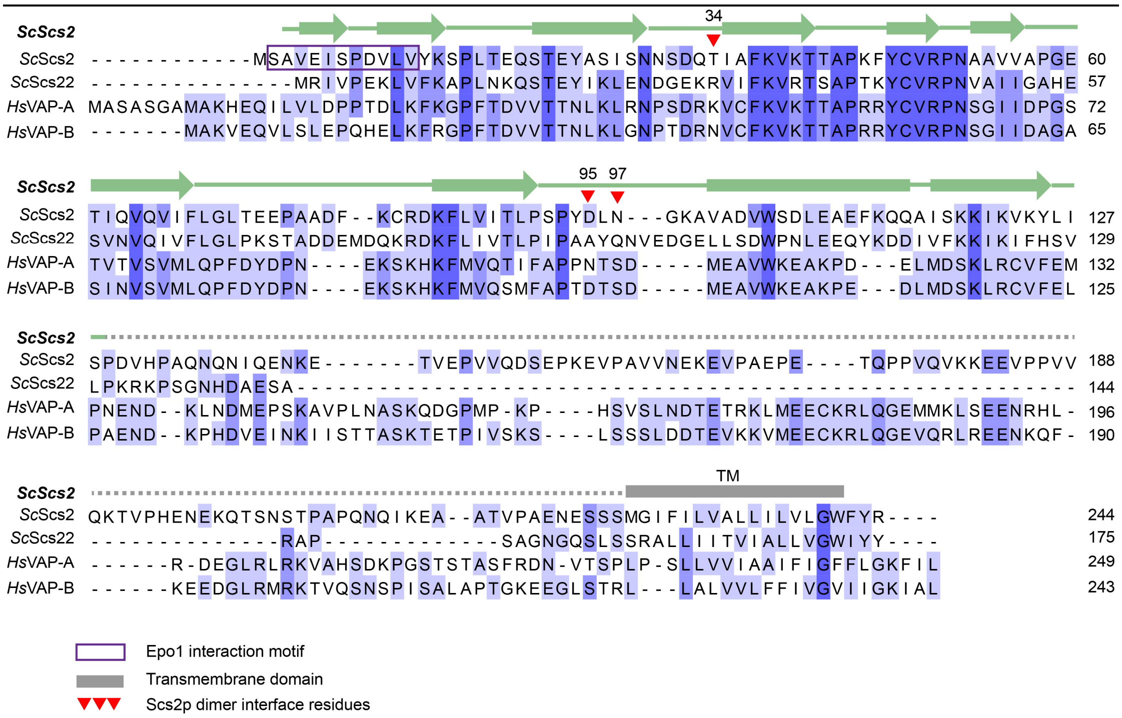

Figure. S2 Sequence alignment of *Sc*Scs2 and other superfamily proteins. Sequence alignment of Scs2, Scs22, Saccharomyces cerevisiae(Sc), Homo sapiens(Hs). The residues with blue, light blue or white backgrounds indicate the identical, conserved or non-conserved residues.

Table S1. Data collection and refinement statistics.

Parameters (Data collection statistics)	Se-Met Epo1-Bem3	Se-Scs2-L86M
**Data collection statistics**		
Cell parameters		
a (Å)	119.4	70.66
b (Å)	119.4	70.66
c (Å)	144.6	56.54
α, β, γ (°)	90.0, 90.0, 90.0	90,90,120
Space group	$P4_12_12$	$P3_22_1$
Wavelength used (Å)	0.9798	0.9798
Resolution (Å)	50.0–3. 95(4.23-3.95)[c]	50.0–2.0(2.13-2.0)[c]
No. of all reflections	756,040 (47,104)	131,646
No. of unique reflections	10,485(1,249)	9,897
Completeness (%)	99.9 (69.2)	99.9 (100)
Average I/σ(I)	10.31 (1.08)	25.2(8.4)
R_{merge}[a] (%)	43.1 (96.1)	11.5(33.9)
**Refinement statistics**		
No. of reflections used (σ(F) > 0)	13,161	10,059
R_{work}[b] (%)	33.9	23.6
R_{free}[b] (%)	39.8	29.6
r.m.s.d. bond distance (Å)	0.04	0.08
r.m.s.d. bond angle (°)	0.834	1.253
Average B-value (Å^2)	95.5	37.4
No. of protein atoms	4,645	978
No. of ligand atoms	0	0
No. of solvent atoms	0	0
Ramachandran plot		
res. in favored regions (%)	92.5	93.5
res. in allowed regions (%)	6.58	5.6
res. in outlier regions (%)	0.91	0.9

[a] $R_{merge} = \Sigma_h\Sigma_l \mid I_{ih}-I_h \mid/\Sigma_h\Sigma_l I_h$ where I_h is the mean of observations I_{ih} of reflection h.

[b] $R_{work} = \Sigma(\|F_p(obs)|-|F_p(calc)\|)/ \Sigma|F_p(obs)|$. R_{free} is an R factor for a pre-selected subset (5%) of reflections that was excluded in the refinement.

[c] Numbers in parentheses are corresponding values for the highest resolution shell.
